# Fermented Lignan-Enriched Soy Beverage Ameliorates the Metabolic Effects of a High-Fat Diet on Female Mice

**DOI:** 10.1021/acs.jafc.4c06947

**Published:** 2025-02-22

**Authors:** Ana Ruiz de la Bastida, Susana Langa, Ángela Peirotén, José Antonio Curiel, Raúl Fernández-González, María Maroto, Juan Arqués, Alfonso Gutiérrez-Adán, José María Landete

**Affiliations:** † Departamento de Tecnología de Alimentos, 54402 Instituto Nacional de Investigación y Tecnología Agraria y Alimentaria, Consejo Superior de Investigaciones Científicas (INIA−CSIC), Madrid 28040, Spain; ‡ Departamento de Reproducción Animal, Instituto Nacional de Investigación y Tecnología Agraria y Alimentaria, Consejo Superior de Investigaciones Científicas (INIA−CSIC), Madrid 28040, Spain

**Keywords:** fermented soy beverage, high-fat diet, isoflavones, flavonoids, lignans, *Bifidobacterium*, fertility

## Abstract

Fermented vegetable beverages have potential beneficial effects on the health associated with the production of bioactive flavonoids and lignans by selected bacterial strains. Here, we studied the effects of a soy beverage and a soy beverage fermented by *Bifidobacterium pseudocatenulatum* INIA P815, both supplemented with lignan extracts, in a female mouse model on a high-fat diet followed for 16 weeks. The high-fat diet induced an increase in adipose tissue and plasma cholesterol as well as modified the fecal microbiota. Mice groups receiving any of the beverages showed a reduction in the mean area of ovarian fat tissue adipocytes and exhibited bioactive flavonoids and lignans in plasma and tissues, accompanied by a higher antioxidant activity in plasma. The group of mice subjected to the fermented beverage also demonstrated a lower increase in plasma cholesterol levels, an increase in short-chain fatty acid production, and higher levels of daidzein, genistein, enterolignans, and herbacetin in the plasma and organs. Moreover, the fertility of the mice that received the fermented beverage was also enhanced, resulting in a higher percentage of blastocysts per female mouse. Therefore, the consumption of the beverage fermented by *B. pseudocatenulatum* INIA P815 could be favoring the health of mice by ameliorating, to some extent, the effects of a high-fat diet.

## Graphical abstract

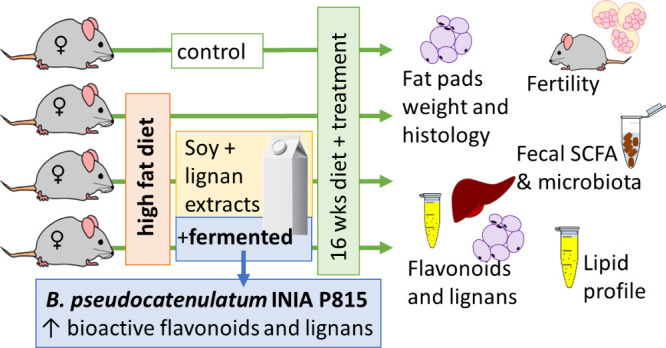

## Introduction

There has been an increase in the manufacturing of vegetable beverages with the aim of meeting the growing demand for plant-based products by consumers.^1^ The main reasons for this demand include a growing awareness among people about the relationship between food and health, environmental protection, and animal rights. Moreover, vegetable beverages are considered a suitable choice in cases of lactose intolerance, poor absorption, allergies to cow’s milk proteins, or hypercholesterolemia.^2^ Among vegetable beverages, a soy beverage stands out due to its greater consumption and its potential beneficial effects on health due to the presence of soy protein, isoflavones, and phytoestrogens associated with the improvement of menopause symptoms, lipid profile, or cardiovascular diseases. Another source of phenolic compounds is flaxseed, with a high content in the phytoestrogen lignans and other compounds with beneficial effects on health.^3^ Among other beneficial effects, phenolic compounds, such as isoflavones and lignans, have great potential in inhibiting the detrimental effects of high-fat-diet-induced obesity.^4,5^

The effect of phenolic compounds in the organisms is highly influenced by its transformation into more bioavailable and bioactive forms, such as isoflavone aglycones, enterolignans, herbacetin, and quercetin.^6^ Like this, a decrease in the risk of hyperlipidemia, atherosclerosis, and cardiovascular diseases by the consumption of soybean products fermented by lactic acid bacteria strains was observed^7,8^ and linked to the increase of aglycone isoflavones and their antioxidant capacities. Certain strains of lactic acid bacteria and bifidobacteria are able to enrich a vegetable beverage in bioactive phytoestrogens through fermentation.^3^ Previous studies have shown that *Bifidobacterium pseudocatenulatum* INIA P815 possesses high glycosidase activity, transforming glycosylated isoflavones and lignans present in a soy beverage or flaxseed.^3,9^ In this way, the administration of a soy beverage fermented by *B. pseudocatenulatum* INIA P815 provided health benefits in lipid profile and fertility in a murine model of menopause.^10^

Based on the results obtained in our previous work,^10^ our objective was to assess the effects of soy beverage enriched with lignan extracts and fermented by *B. pseudocatenulatum* INIA P815 on a murine model of obesity. Therefore, lignan-extract-enriched soy beverages, with or without the fermentation of *B. pseudocatenulatum* INIA P815, were tested in CD1 female mice, which have been described to be prone to diet-induced obesity,^11,12^ on a high-fat diet followed for 16 weeks. A diet-induced obesity model was chosen as a better approximation to the common obesity state in humans^13^ to test the effects of a daily intake of enriched soy beverages. Therefore, the effects of both beverages were compared in terms of fat accumulation, lipid profile, glucose tolerance, short-chain fatty acid (SCFA) production, and fecal microbiota profile. The presence of bioactive flavonoids and lignans in the plasma and liver of the mice was also analyzed as well as the influence of the beverages on their fertility.

## Materials and Methods

### Bacterial Strain and Culture Conditions

*B. pseudocatenulatum* INIA P815 was cultivated at 37 °C in MRS broth (BD, Becton, Dickinson & Co., Le Pont de Claix, France) supplemented with 0.5 g/L l-cysteine (Merck KGaA, Darmstadt, Germany) (MRS-cys) under anaerobic conditions (10% H_2_, 10% CO_2_, and 80% N_2_, Whitley DG250 anaerobic workstation, Don Whitley Scientific, Ltd., Shipley, U.K.).

### Soy Beverage and Fermented Soy Beverage Preparation and Analysis

The commercial soy beverage Vital (13% soy, DIA, Madrid, Spain) was supplemented with lignan extracts from flax (10 g/L, 20% secoisolariciresinol diglucoside, LinumLife EXTRA, Frutarom Netherlands BV, Veenendaal, Netherlands) and autoclaved to 121 °C for 1 min to obtain the supplemented soy beverage. After cooling to 37 °C, this supplemented soy beverage was inoculated with *B. pseudocatenulatum* INIA P815 according to Ruiz de la Bastida et al.^9^ at an initial concentration of 6.5–7.0 log CFU/mL and incubated for 24 h at 37 °C under anaerobic conditions to obtain a fermented soy beverage with 8.5 ± 0.4 log CFU/mL of the bifidobacteria. Both fermented and non-fermented supplemented beverages were aseptically aliquoted and stored at −35 °C.

Bacterial counts and pH determinations were performed before and after the fermentation as described by Ruiz de la Bastida et al.^10^ Briefly, serial dilutions of the beverages were plated in MRS agar supplemented with cysteine and incubated at 37 °C under anaerobic conditions, and pH was measured with a Crison pH meter (model GPL 22, Crison Instruments, Barcelona, Spain). The content in flavonoids and lignans in both beverages was analyzed by high-performance liquid chromatography–electrospray ionization mass spectrometry (HPLC–ESI/MS, Beckman System Gold, Beckman Coulter, Inc., Fullerton, CA, U.S.A.) according to Gaya et al.^14^ The ferric reducing ability of plasma (FRAP) was estimated in both beverages according to Ruiz de la Bastida et al.^10^

### Animals, Experimental Groups, and Study Design

Animal experiments were conducted by following European legislation. All study protocols were approved by the Ethical Committee on Animal Experimentation of INIA (Madrid, Spain) and were registered on Dirección General de Agricultura y Ganadera de la Comunidad de Madrid, Spain (PROEX 076.6/21).

CD1 female mice (*n* = 72), aged 5 months, were housed in an animal facility with a controlled temperature (23 ± 1 °C) and photoperiod (cycle of 14 h light/10 h darkness). This age was chosen because at 5 months CD1 female mice have already undergone sexual maturation and the phase of exponential growth and are close to beginning reproductive function failures that occur for this strain at 7–8 months of age.^15^ Mice were fed either a standard diet (*n* = 18, distributed in 3 cages), with a basic diet low in phytoestrogens (SAFE 150, Scientific Animal Food & Engineering, Augy, Bourgogne Franche-Comté, France), or a high-fat diet (*n* = 54, SAFE U8954 Version 204, Scientific Animal Food & Engineering). The high-fat diet contains 24% crude fat, with 45.9% of its energy content coming from lipids, while the standard diet contains 4.8% crude fat, with 12.6% of its energy content coming from lipids. Mice fed with a standard diet were considered the control group (CNT) and did not receive any beverage. Mice fed with a high-fat diet were subdivided into three subgroups (*n* = 18, distributed in 3 cages) depending upon the administered beverage: control subgroup not receiving any beverage (HFD), subgroup receiving lignan-supplemented soy beverage (SB), and subgroup receiving the fermented version of the beverage (FSB). Beverages were administered daily for 16 weeks in drinking bottles with 24 mL of the respective beverage, resulting in an average consumption of 4 mL per mouse and day. The animal body weight and food intake were controlled weekly. Blood samples were collected, from each group, before the start of the experiment (T0) and during the last week before the fertility assessment and sacrifice (FT). They were taken 1 h after the beverage administration, by submandibular puncture, in ice-cold tubes containing heparine and immediately centrifuged for 10 min at 1000*g* and 4 °C. Plasma samples were stored at −80 °C until analyses.

Female mice were housed with CD1 males along the last week of the experiment. Mice continued to receive their corresponding diet and beverage, and females were checked for copulatory plugs every morning. Any female with a copulatory plug was weighted and sacrificed by cervical dislocation; the rest of the female mice were sacrificed on the last day of the experiment. Mice length was measured after sacrifice. Ovarian, inguinal, and interscapular white adipose tissues (WATs), brown adipose tissue (BAT) between the scapulars, and the liver were dissected from each animal and weighted. Portions of the liver and ovarian WAT were immediately frozen in liquid nitrogen and stored at −80 °C until analysis. Oocytes in metaphase II and zygotes were collected from the ampulla of the oviduct and incubated with hyaluronidase (300 mg/mL) to remove cumulus cells in M2 medium tempered at 37 °C. They were counted and cultured for 4 days at 37 °C, 5% CO_2_, and humidity in droplets of 30 μL of KSOM [EmbryoMax Advanced KSOM Embryo Medium (MR-101-D)] covered by oil until the blastocyst stage to check their development.

### Glucose Tolerance Test and Lipid Profile and Antioxidant Activity of Plasma

Three weeks before the sacrifice, the glucose tolerance test was carried out individually. Mice were fasted overnight (16 h) and then intraperitoneally injected with a sterile 20% glucose solution in phosphate-buffered saline (PBS) at a dose of 1.5 g/kg of body mass. Measures of blood glucose levels were taken using a glucometer and strips (Glucomen Aerosensor, Menarini, Diagnosticos, Madrid, Spain) from a drop of blood from the tip of the tail before glucose injection (0 point) and at 15, 30, 60, and 120 min after injection. Food and water were immediately returned after the last measurement was taken.

For the lipid profile and antioxidant capacity analysis, plasma samples were obtained from a pool of blood extracted from mice within the same cage at T0 and FT (3 samples per group of condition). Total cholesterol in plasma was measured using the Cholesterol Fluorometric Assay Kit (10007640, Cayman Chemical, Ann Arbor, MI, U.S.A.) following the manufacturer’s instructions; plasma samples were diluted 400 times. A Spark 20M microplate reader (Tecan, Switzerland) was used for the determination of total cholesterol, exciting the samples at 540 nm and detecting the fluorescence at 595 nm. Total triglycerides in plasma were measured with the Triglyceride Colorimetric Assay Kit (10010303, Cayman Chemical, Ann Arbor, MI, U.S.A.); plasma samples were diluted 2 times. Mouse high-density lipoprotein cholesterol (HDL-C) and low-density lipoprotein cholesterol (LDL-C) in plasma were measured with the Mouse HDL (High-Density Lipoprotein) ELISA Kit (EM1113, FineTest, Wuhan, China) and the Mouse LDL (Low-Density Lipoprotein) ELISA Kit (EM1184, FineTest, Wuhan, China), respectively, following the manufacturer’s instructions; plasma samples were diluted 200 times in both cases. A Multiskan Spectrum spectrophotometer (Thermo Fisher Scientific, Waltham, MA, U.S.A.) was used for the total triglycerides (absorbance lecture at 540 nm) and HDL-C and LDL-C (absorbance lecture at 450 nm) determination. Total cholesterol, total triglycerides, and HDL-C and LDL-C concentrations were calculated using the equation obtained from the linear regression of the standard curve for each parameter. To measure the antioxidant capacity, the FRAP assay was estimated in plasma samples at FT according to Ruiz de la Bastida et al.^10^

### Identification and Quantification of Flavonoids and Lignans in Mouse Plasma, Liver, and Ovarian Fat

The flavonoids and lignans were extracted from plasma samples (obtained as explained in the previous section) following the method proposed by Timan et al.,^16^ with the modifications established by Ruiz de la Bastida et al.^10^ The extraction of flavonoids and lignans from mouse liver and ovarian fat was performed following the method proposed by Gilani et al.,^17^ with modifications indicated previously by Ruiz de la Bastida et al.^10^ Extracted flavonoids and lignans were later analyzed by means of HPLC–ESI/MS according to Gaya et al.^18^ External calibration curves were employed for quantification using HPLC-grade standard compounds. Daidzin, genistein, daidzein, genistein, dihydrodaidzein (DHD), dihydrogenistein (DHG), kaempferol, naringenin, quercetin, secoisolariciresinol (SECO), matairesinol, pinoresinol, enterodiol (END), and enterolactone (ENL) were purchased from Merck KGaA (Darmstadt, Germany). Herbacetin was purchased from Extrasynthese (Genay, France). *O*-Desmethylangolensin (*O*-DMA) and 6-hydroxy-*O*-desmethylangolensin (6-OH-*O*-DMA) quantification was done with the calibration curves of the most similar compounds DHD and DHG. Stock solutions of standards (10 mg/mL) were prepared in dimethyl sulfoxide (DMSO, Merck KGaA).

### Ovarian WAT Histological Analysis

A portion of the ovarian WAT was fixed using 4% formaldehyde diluted in PBS, embedded in paraffin, and then sectioned for histological analysis. Hematoxylin and eosin (H&E) staining was performed according to the standard procedure. Digital images at 10× augmentation of H&E-stained tissue sections were taken and analyzed with the ImageJ program using the Adiposoft plugin^19^ for adipocyte size determination. The average adipocyte size was expressed as the average cross-sectional area per cell and calculated based on at least 100 adipocytes per digital image. Fat pads of five or six mice from each experimental group were analyzed, measuring four different images from two different tissue sections of each of the fat samples.

### SCFA Determination in Feces

Fresh stool samples were taken in pools from every cage before mating and sacrifice, kept in ice, and processed shortly after collection to avoid SCFA lost. Stools were homogenized, mixed with phosphoric acid buffer (0.5%, 0.1 g/mL), and kept for 24 h at −80 °C. Later, they were thawed, vortexed for 2 min, and centrifuged (14 000 rpm, 10 min, 4 °C). Supernatants were collected and maintained at −20 °C until analysis. Acetic, propionic, isobutyric, butyric, isovaleric, valeric, caproic, and heptanoic acids were analyzed in an Agilent 6890A gas chromatography (GC) system (Agilent Technologies, Palo Alto, CA, U.S.A.), equipped with a flame ionization detector (FID) and a G2613A automatic injector, according to García-Villalba et al.^20^

### Fecal Microbiota Characterization

Fresh stool samples were collected from four mice of each group independently at the end of the experiment and frozen at −80 °C immediately until analysis by next-generation sequencing (Secugen SL, Madrid, Spain). The analysis was performed on independent samples. After thawing, the samples were homogenized and resuspended in a stabilizing solution. DNA was extracted by severe mechanical disruption with zirconia beads and column purification. The RNA 16S gene was amplified by polymerase chain reaction (PCR) with primers 27Fv1-5′AGRGT­TYGAT­YHTGG­CTCAG3′ and 1492R-5′CGGTT­ACCTT­GTTAC­GACTT3′, with independent labels for each sample. The purified products of the amplification reactions were mixed in equimolar amounts. A tag was added to this set of amplicons by ligation with the SQK-NBD114.24 kit, and this product was sequenced with Oxford Nanopore Technology MinION FLO-MIN114 R10.4.1 in conditions of maximum precision. The reads were reanalyzed with very high-quality parameters and filtered by size (1300–1800 nt), and an average of 20 000 reads per sample was obtained. Reads were processed with VSEARCH software to remove chimera generated during amplification. Taxonomic assignment of reads was done with EMU software.^21^ Species with fewer than 10 reads were not reported.

### Statistical Analysis

Parametric statistical analyses were performed with the analysis of variance (ANOVA), using a general linear model (GLM), and by Tukey’s multiple range test. Non-parametric statistical analyses were carried out using the Kruskal–Wallis test and Mann–Whitney *U* test. Data related to fertility were analyzed by three-way ANOVA, and subsequently, those data with significant differences were transformed through the arcsine of the square root and analyzed by an unpaired *t* test. Data were expressed as the mean ± standard deviation (SD), and differences were considered significant at *p* < 0.05. The SPSS Statistics 22.0 software (IBM Corp., Armonk, NY, U.S.A.) and the GraphPad Prism version 8 software (Dotmatics, Bishop’s Stortford, U.K.) were used for the statistical analysis.

## Results

### Soy Beverage and Fermented Soy Beverage Analysis

The glycosylated isoflavones daidzin and genistin were predominant in the supplemented soy beverage (Table 1), while in the fermented beverage, they were completely metabolized into their aglycones daidzein and genistein. DHD and DHG were found in both beverages always in concentrations lower than 0.1 μM. The fermented beverage showed the presence of *O*-DMA and 6-OH-*O*-DMA in concentrations between 0.1 and 0.2 μM. High concentrations of SECO and herbacetin were observed in the fermented beverage, whereas herbacetin did not appear in the non-fermented beverage and SECO was detected at low concentrations (Table 1). Other flavonoids, such as naringenin and kaempferol, were found in concentrations between 0.5 and 4 μM in both beverages, with the concentrations always being higher in the fermented beverage. Quercetin, matairesinol, and pinoresinol appeared only in the fermented beverage. In the same way, the fermented beverage presented a higher antioxidant activity in contrast to the non-fermented beverage (39.27 ± 1.38 and 29.13 ± 0.89 mg/mL TROLOX, respectively).

**Table 1:** Flavonoid and Lignan Contents in Supplemented Soy Beverage or Fermented Supplemented Soy Beverage^a^

	supplemented soy beverage	fermented supplemented soy beverage
daidzin	145.323 ± 24.191	12.053 ± 1.278
genistin	318.381 ± 7.498	16.403 ± 3.561
daidzein	6.553 ± 0.756	277.765 ± 29.829
genistein	18.454 ± 2.682	366.892 ± 56.857
DHD	0.074 ± 0.048	0.053 ± 0.018
DHG	0.032 ± 0.013	0.042 ± 0.011
*O*-DMA	0.063 ± 0.041	0.187 ± 0.104
6-OH-*O*-DMA	0.045 ± 0.015	0.138 ± 0.071
SECO	12.925 ± 0.959	652.746 ± 18.892
matairesinol	nd	5.674 ± 2.309
pinoresinol	nd	2.455 ± 1.772
END	nd	nd
ENL	nd	nd
herbacetin	nd	346.890 ± 32.614
naringenin	0.553 ± 0.281	2.439 ± 1.560
kaempferol	0.782 ± 0.423	4.560 ± 0.957
quercetin	nd	2.204 ± 0.850

a Values are the mean ± SD. nd = not detected. DHD, dihydrodaidzein; DHG, dihydrogenistein; *O*-DMA, *O*-desmethylangolensin; 6-OH-*O*-DMA, 6-hydroxy-*O*-desmethylangolensin; SECO, secoisolariciresinol; END, enterodiol; and ENL, enterolactone.

### Effect of the Beverages on Body, Liver, and Fat Weights

The 72 mice were distributed arbitrarily among the different experimental groups, which did not show significant differences in their initial body weight. A similar dynamic of body weight increment was observed in all groups, and no significant differences were observed at the end of the experiment (panels A and B of Figure 1). Likewise, the four groups did not present significant differences in daily calorie intake per mouse (*p* < 0.05) (Figure 1C). Even though no significant differences were observed in the Lee index (Figure 1D) and the liver relative weight (Figure 1E), significantly higher ovarian, inguinal, and interscapular WAT relative weights were found in the groups that received the high-fat diet (panels F, G, and H of Figure 1). In addition, the relative weight of BAT did not present significant differences between groups (Figure 1I).

**Figure 1:**
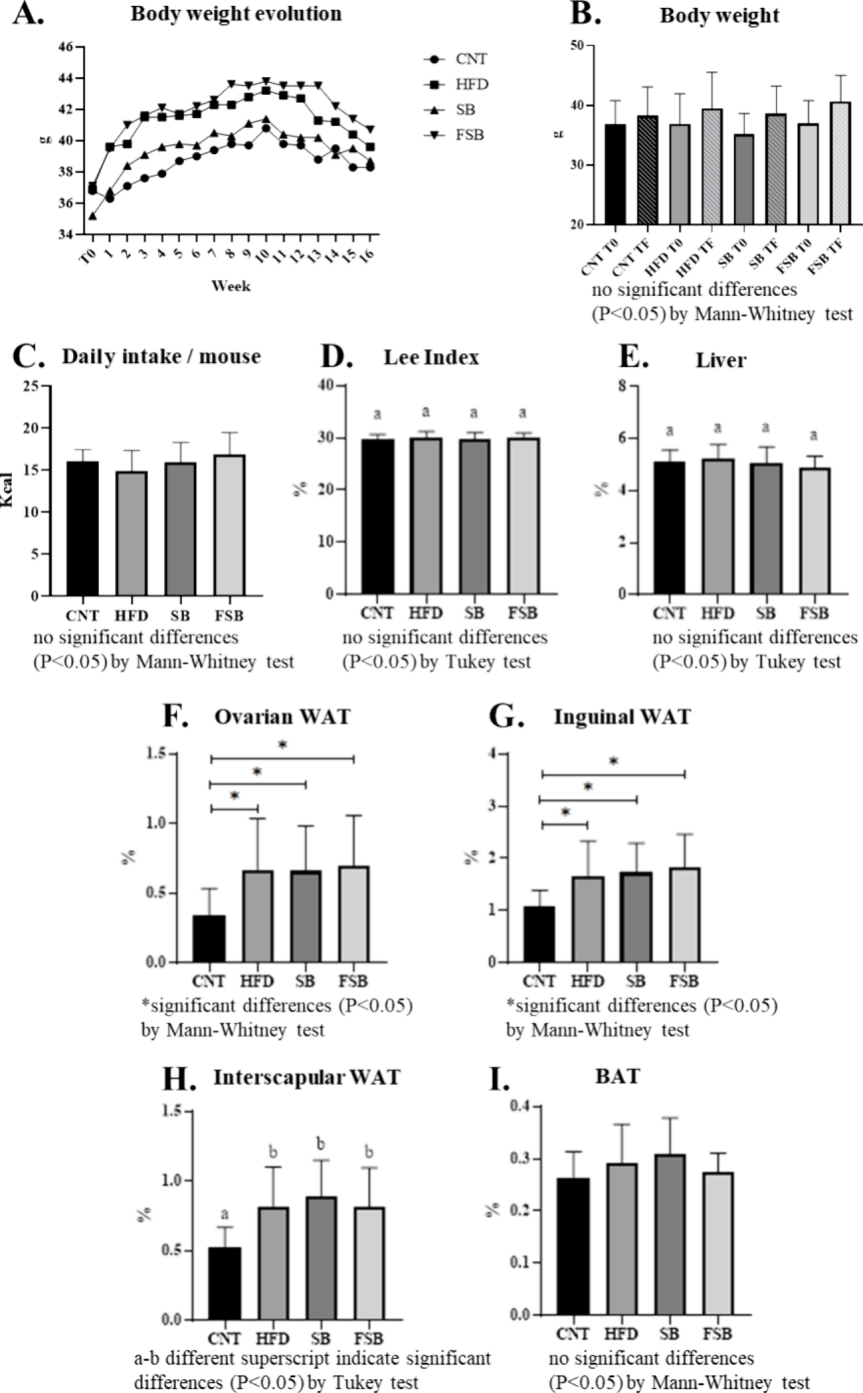
Body, liver, and fat weight of mice: control (CNT) and under a high-fat diet (HFD) receiving supplemented soy beverage (SB) or fermented supplemented soy beverage (FSB). (A) Body weight evolution, (B) daily kcal intake per mouse, (C) Lee index [(3 square root body weight (g)/nasoanal length (cm)) × 1000], (D) relative liver weight, (E) relative ovarian white adipose tissue (WAT) weight, (F) relative inguinal WAT weight, (G) relative interscapular WAT weight, and (H) relative weight of the interscapular brown adipose tissue (BAT). Values are the mean ± SD; 12 mice per group.

### Effect of Fermented and Non-fermented Beverages on the Reproductive Biology of Mice

After natural mating, no differences were observed in the number of plugged females among groups (Figure 2A). With regard to the average number of hemorrhagic follicles per female mouse, a parameter that allows the measurement of ovulation, a slight increase in the number of ovulated oocytes was seen in the FSB group compared to the rest of the groups, mainly in mice that did not show copulatory plugs, although the differences were not significant (Figure 2B). Finally, in the percentage of blastocysts per female mouse, a striking increase was observed in the FSB group compared to the rest, with the difference with the SB group being statistically significant (*p* = 0.0462) (Figure 2C).

**Figure 2:**
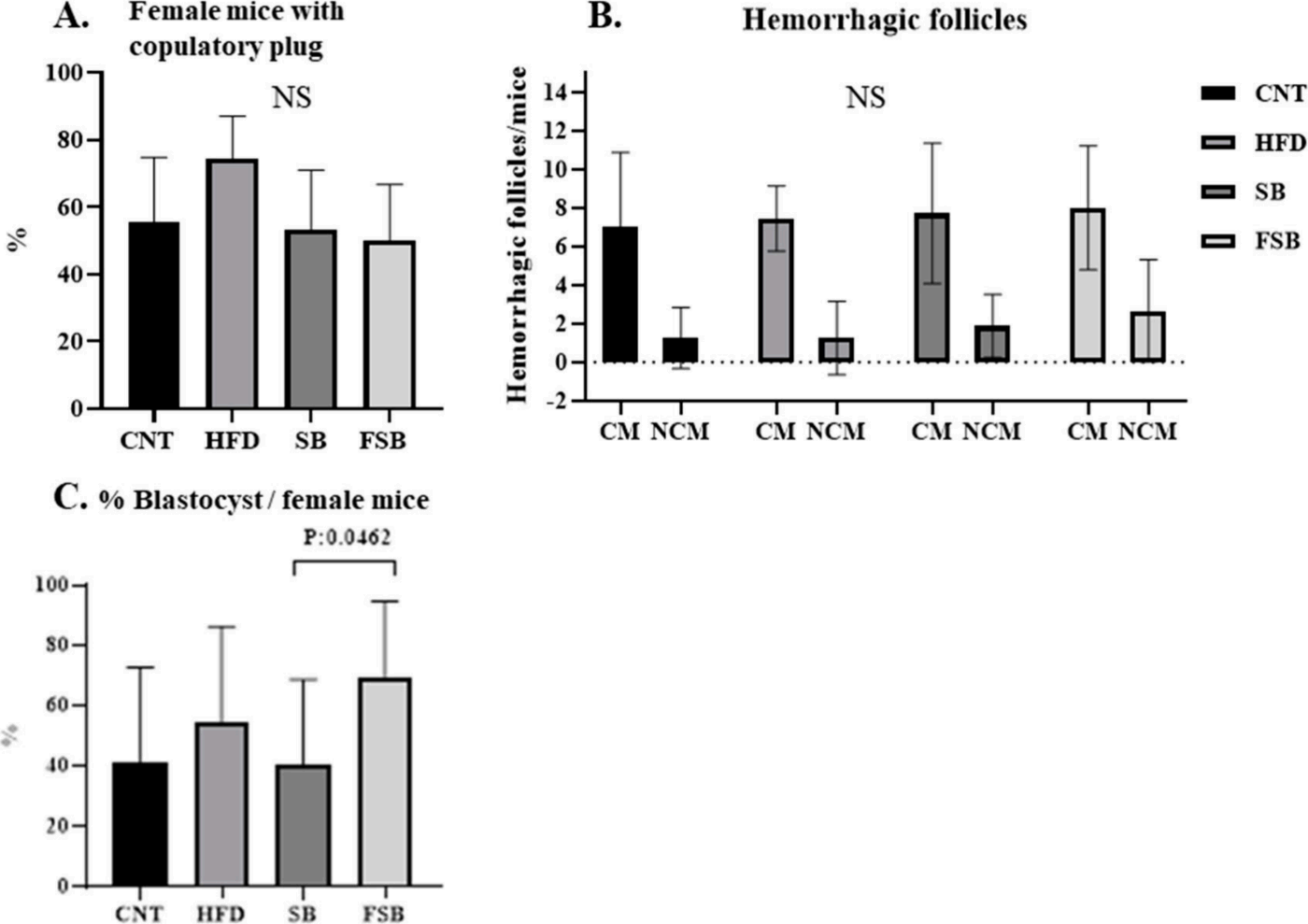
Fertility data on the four mice groups: control (CNT), control under a high-fat diet (HFD), receiving a high-fat diet and supplemented soy beverage (SB), or receiving a high-fat diet and supplemented with fermented supplemented soy beverage (FSB). (A) Percentage of female mice presenting a copulatory plug per group, (B) hemorrhagic follicles per female mice, with the graph distinguishing between mice with (CM) and without (NCM) a copulary plug in each group, and (C) percentage of blastocyst per female mice. Values are the mean ± SD; 12 mice per group. Differences were considered significant at *p* < 0.05 by the unpaired *t* test. NS = not statistically significant.

### Effect of SB and FSB Beverages on the Lipid Profile and Glucose Tolerance

Different biochemical parameters related to the lipid profile were analyzed in mice plasma. While in the CNT group, the total cholesterol at FT maintained its levels compared to those at T0, and in the groups that followed a high-fat diet, the levels increased significantly (Figure 3A). It should be noted that total cholesterol in the FSB group was statistically lower than the other groups under a high-fat diet. With regard to the total triglycerides, these suffered a significant decrease in the three groups with a high-fat diet compared to the CNT group (Figure 3B). The same occurred with HDL-C, which suffered a significant decrease in groups receiving a high-fat diet compared to the CNT group (Figure 3C). Finally, in the levels of LDL-C, the three groups with a high-fat diet did not obtain significant differences compared to the values at T0 (Figure 3D). On the other hand, no significant differences were found between the four groups in the glucose tolerance results (panels E and F of Figure 3).

**Figure 3:**
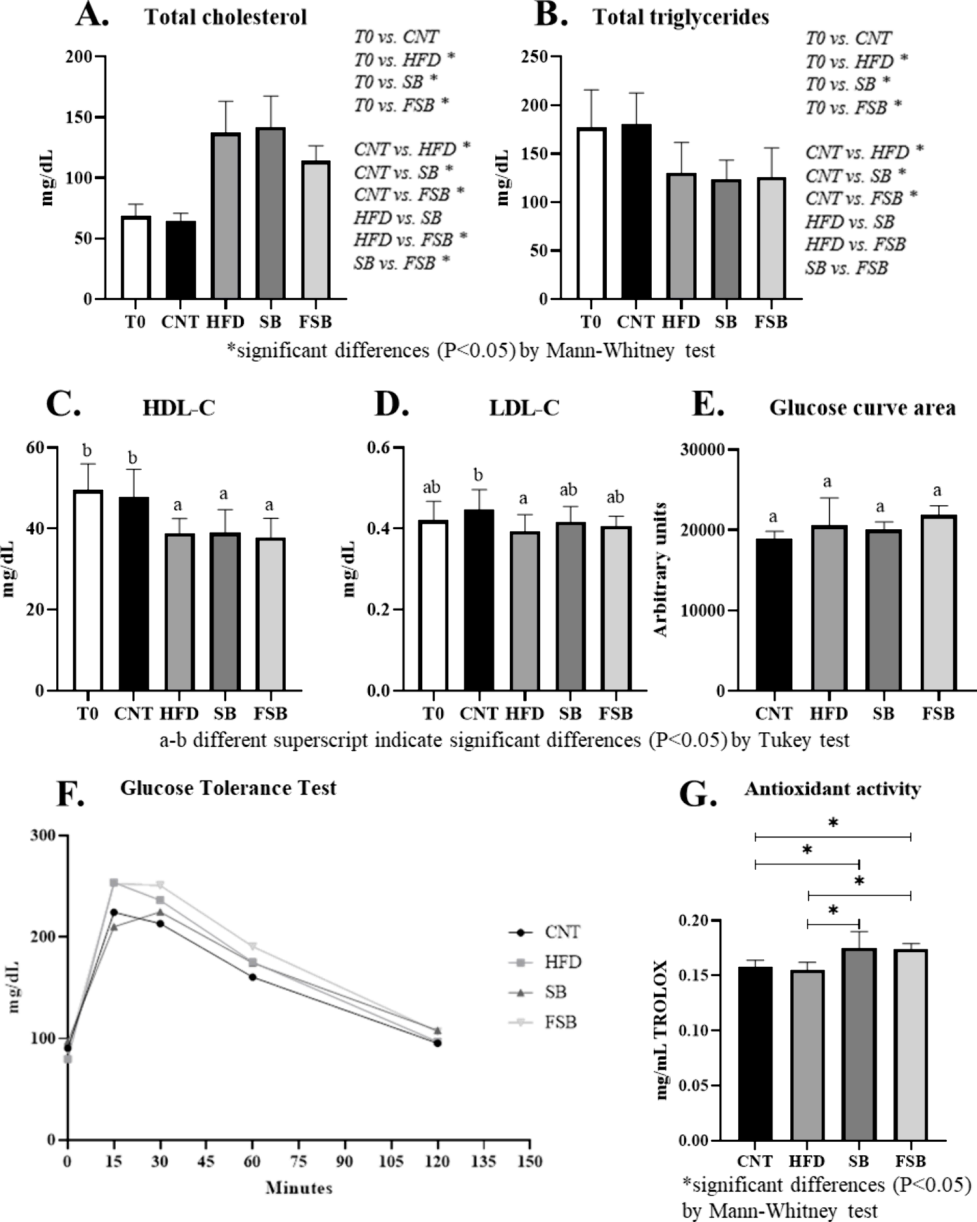
Influence of the high-fat diet and beverages on the lipid profile, plasma antioxidant activity, and glucose tolerance test of the different mice groups: control (CNT; *n* = 3) and under a high-fat diet (HFD, *n* = 3) receiving supplemented soy beverage (SB; *n* = 3) or fermented supplemented soy beverage (FSB; *n* = 3). The initial time (T0; *n* = 12) is also included. (A) Total cholesterol in mice plasma, with statistical differences between groups (two by two) indicated on the right, (B) total triglycerides in mice plasma, with statistical differences between groups (two by two) indicated on the right, (C) HDL-C in mice plasma, (D) LDL-C in mice plasma, (E) glucose tolerance (measured as the glucose curve area), (F) glucose tolerance test results, and (G) antioxidant activity of mice plasma. Values are the mean ± SD.

### Plasma Antioxidant Activity and Presence of Flavonoids and Lignans in Plasma, Liver, and Ovarian WAT

The antioxidant activity measured in mice plasma was significantly higher in the SB and FSB groups compared to the CNT and HFD groups without a beverage (Figure 3G), while no differences were found between SB and FSB groups. Likewise, the CNT and HFD groups, which did not intake any type of soy beverage, did not present flavonoids or lignans in plasma, liver, and ovarian WAT (Table 2).

**Table 2:** Flavonoid and Lignan Contents in Mice Plasma and Liver of the Different Mice Groups, Control (CNT) and under a High-Fat Diet (HFD) Receiving Supplemented Soy Beverage (SB) or Fermented Supplemented Soy Beverage (FSB)^a^

	CNT	HFD	SB	FSB
	Plasma (μM)			
daidzein	nd^a^	nd^a^	0.449 ± 0.141^b^	1.456 ± 0.116^c^
genistein	nd^a^	nd^a^	1.094 ± 0.180^b^	2.404 ± 0.448^c^
DHD	nd^a^	nd^a^	0.043 ± 0.021^b^	0.036 ± 0.024^ab^
DHG	nd^a^	nd^a^	nq	nq
*O*-DMA	nd^a^	nd^a^	0.034 ± 0.018^a^	0.026 ± 0.024^a^
6-OH-*O*-DMA	nd^a^	nd^a^	nq	nq
SECO	nd^a^	nd^a^	0.055 ± 0.013^b^	0.102 ± 0.005^c^
END	nd^a^	nd^a^	0.093 ± 0.029^b^	0.098 ± 0.012^b^
ENL	nd^a^	nd^a^	0.167 ± 0.043^b^	0.201 ± 0.032^b^
herbacetin	nd^a^	nd^a^	0.034 ± 0.034^a^	0.615 ± 0.115^b^
naringenin	nd^a^	nd^a^	nq	nq
kaempferol	nd^a^	nd^a^	nq	nq
	Liver (μmol/g)			
daidzein	nd^a^	nd^a^	1.578 ± 0.534^b^	5.534 ± 0.445^c^
genistein	nd^a^	nd^a^	0.527 ± 0.135^a^	3.766 ± 0.791^b^
DHD	nd^a^	nd^a^	nq	nq
DHG	nd^a^	nd^a^	nq	nq
*O*-DMA	nd^a^	nd^a^	nq	nq
6-OH-*O*-DMA	nd^a^	nd^a^	nq	nq
SECO	nd^a^	nd^a^	0.837 ± 0.200^b^	0.689 ± 0.155^b^
END	nd^a^	nd^a^	0.102 ± 0.151^a^	0.251 ± 0.122^a^
ENL	nd^a^	nd^a^	1.042 ± 0.368^b^	2.539 ± 0.687^c^

a Values are the mean ± SD. nd = not detected. nq = not quantified. Different superscript letters (a–c) indicate statistically significant differences (*p* < 0.05) by Tukey’s test. Statistical analysis of the concentration of compounds in plasma and liver was done separately. DHD, dihydrodaidzein; DHG, dihydrogenistein; *O*-DMA, *O*-desmethylangolensin; 6-OH-*O*-DMA, 6-hydroxy-*O*-desmethylangolensin; SECO, secoisolariciresinol; END, enterodiol; and ENL, enterolactone.

The plasma from the SB and FSB groups showed the presence of isoflavone aglycones as well as other secondary compounds of isoflavone metabolism (DHD, DHG, *O*-DMA, and 6-OH-*O*-DMA), other flavonoids (herbacetin, naringenin, and kaempferol), and lignans (SECO, END, and ENL). Daidzein, genistein, SECO, and herbacetin were significantly higher in the plasma of the FSB group than in the SB group, with the concentration of herbacetin being 18 times higher in the FSB group (Table 2).

As for the tissues analyzed, liver and ovarian WAT, they showed the presence of the same flavonoids and lignans observed in plasma in the SB and FSB groups, with the exception of herbacetin, kaempferol, and naringenin, which were not present in any of the tissues. Levels of compounds in ovarian WAT were very low and non-quantifiable. In liver, levels of daidzein, genistein, and ENL were higher in the FSB group than in the SB group and also surpassed the concentrations recorded in plasma (Table 2).

### Ovarian WAT Histology

The size of the adipocytes increased considerably in the three groups that received the high-fat diet compared to the CNT group, with this increase being statistically significant (Figure 4). Also, the values of the adipocyte area of the SB and FSB groups were statistically lower than those of the HFD group not receiving any of the beverages. Mice that received the fermented beverage showed the lowest mean adipocyte area compared to the other two groups with a high-fat diet.

**Figure 4:**
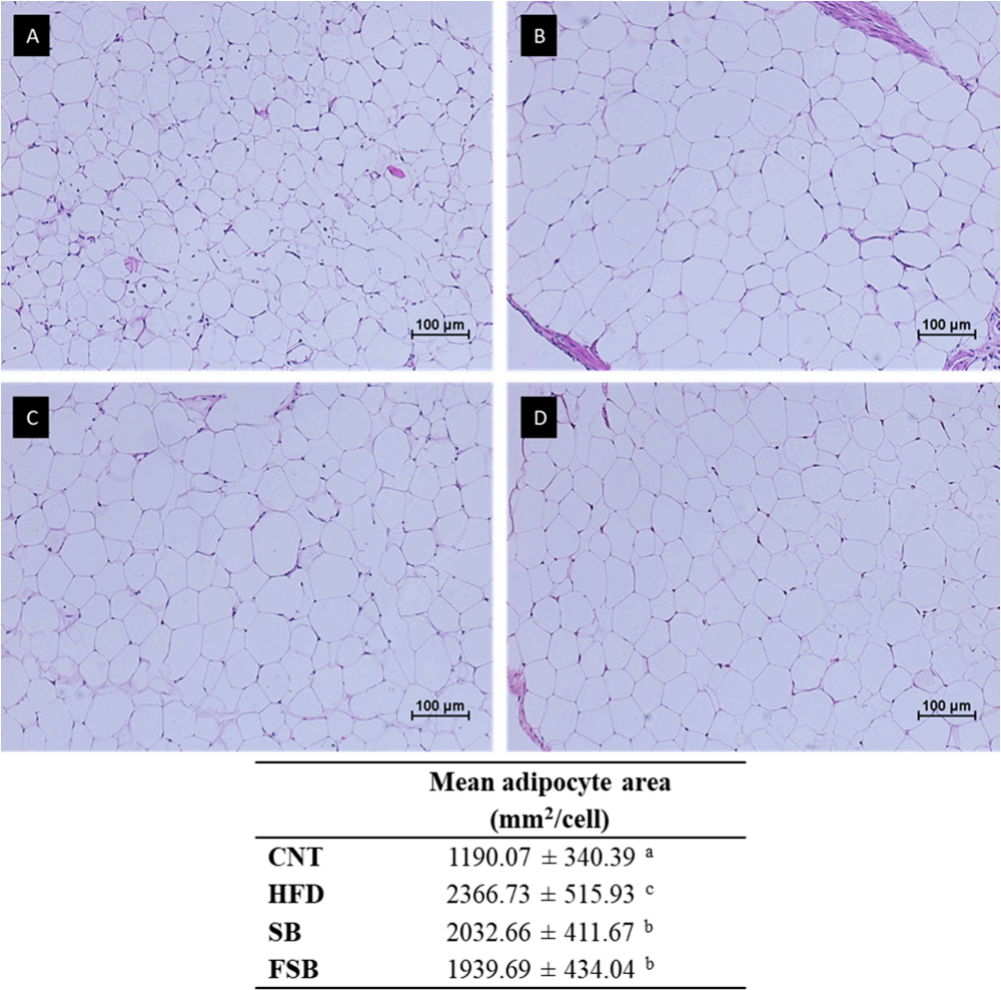
Representative photomicrographs (10× augmentation) of cross sections after H&E staining of the ovarian fat pads. (A) Normal diet group (CNT), (B) high-fat diet group (HFD), (C) high-fat diet + soy beverage group (SB), and (D) high-fat diet + fermented soy beverage group (FSB). Mean adipocyte area in the four groups. Values are the mean ± SD; 6 mice per group. Different superscript letters (a–c) indicate statistically significant differences (*p* < 0.05) by Tukey’s test.

### SCFA Analysis in Stool Samples at the End of the Experiment

Those groups under a high-fat diet (HFD, SB, and FSB) suffered a significant decrease in the total SCFA levels compared to those of the CNT group (Figure 5). This was reflected in the three main SCFAs: acetic acid, propionic acid, and butyric acid. However, among the groups under a high-fat diet, FSB was the group that registered the highest levels in all of the SCFA analyzed, with the exception of caproic acid. Therefore, the levels of total SCFA, acetic acid, and isobutyric acid in the FSB group were significantly higher than those of the SB group. Concerning isobutyric acid, mice in the FSB groups achieved levels not significantly different from the CNT group and significantly higher than those of the other two groups receiving a high-fat diet (HFD and SB). Valeric and isovaleric acids reached very similar levels in all groups, regardless of diet. While caproic acid was not detected in the CNT group, the groups under a high-fat diet registered low amounts of this SCFA. Heptanoic acid was not detected in any of the four groups.

**Figure 5:**
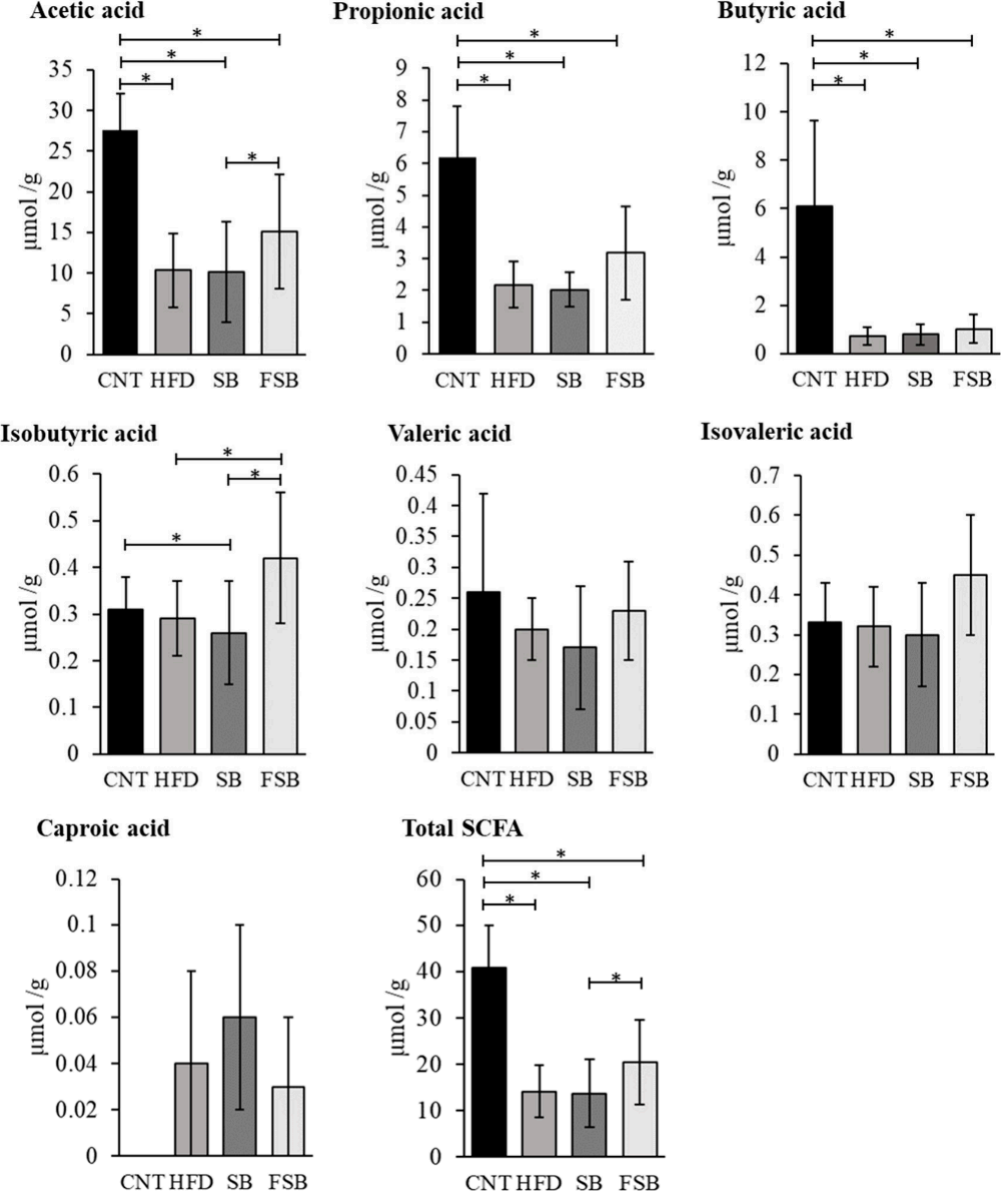
SCFA analyzed in fresh stool samples at a final time in the mice control group (CNT; *n* = 3) and groups under a high-fat diet (HFD; *n* = 3) and receiving supplemented soy beverage (SB; *n* = 3) or fermented supplemented soy beverage (FSB; *n* = 3). Concentration of SCFA (μmol/g) per group. Non-parametric statistical analysis of the data. ∗ indicates statistically significant differences (*p* < 0.05) by Mann–Whitney tests. Any paired comparison without ∗ shows no significant difference.

### Metagenomic Assay of Fecal Microbiota

The impact on the fecal microbiota of the lignan-enriched soy beverages on a high-fat diet mice model was explored after 16 weeks of treatment. The analysis of the microbial diversity, calculated as Shannon index, showed a similar diverseness between the samples analyzed, with the fecal microbiota from SB being the ones that exhibited the least variance (Figure 6A). The relative abundance of the bacterial communities was also analyzed.

**Figure 6:**
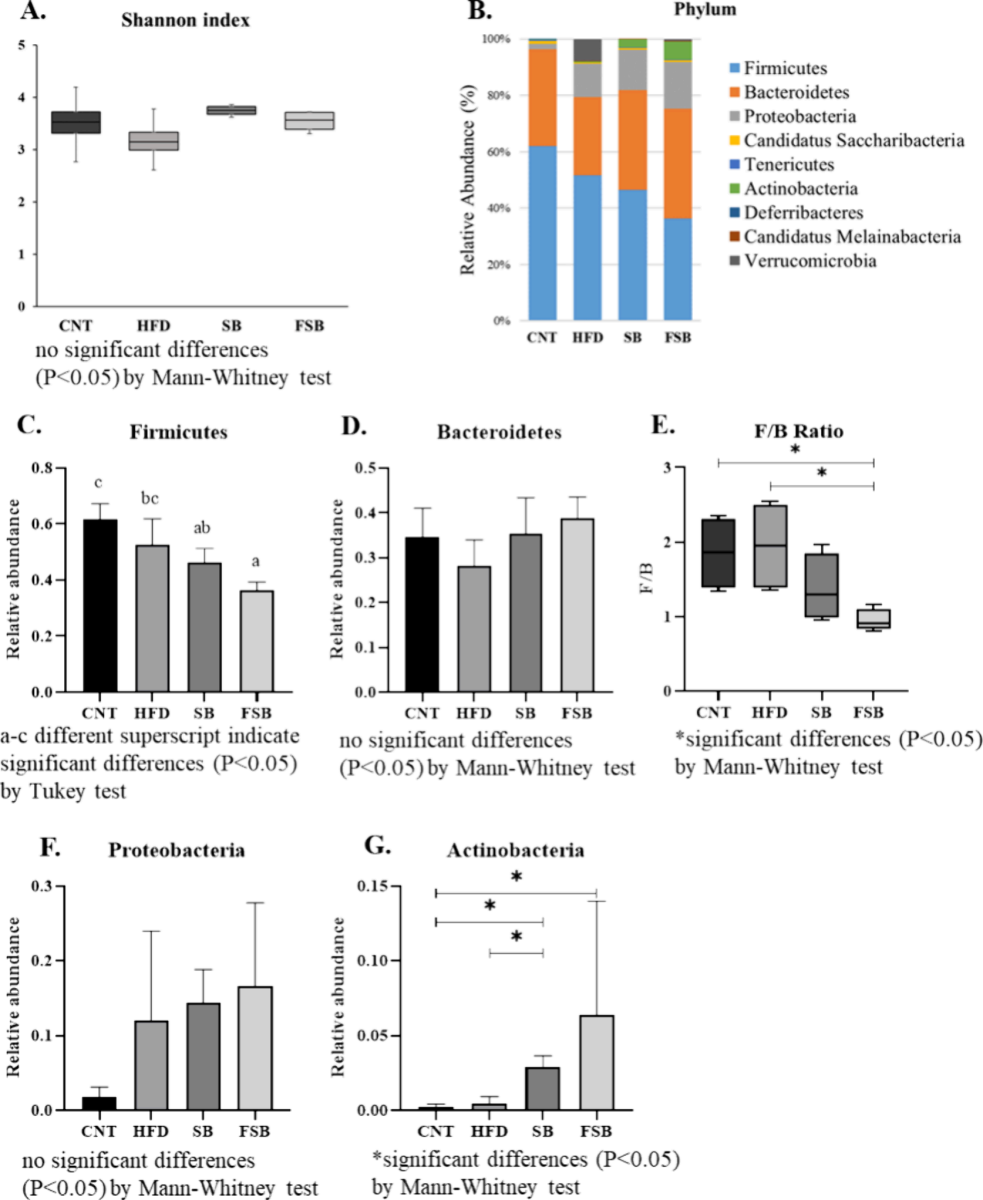

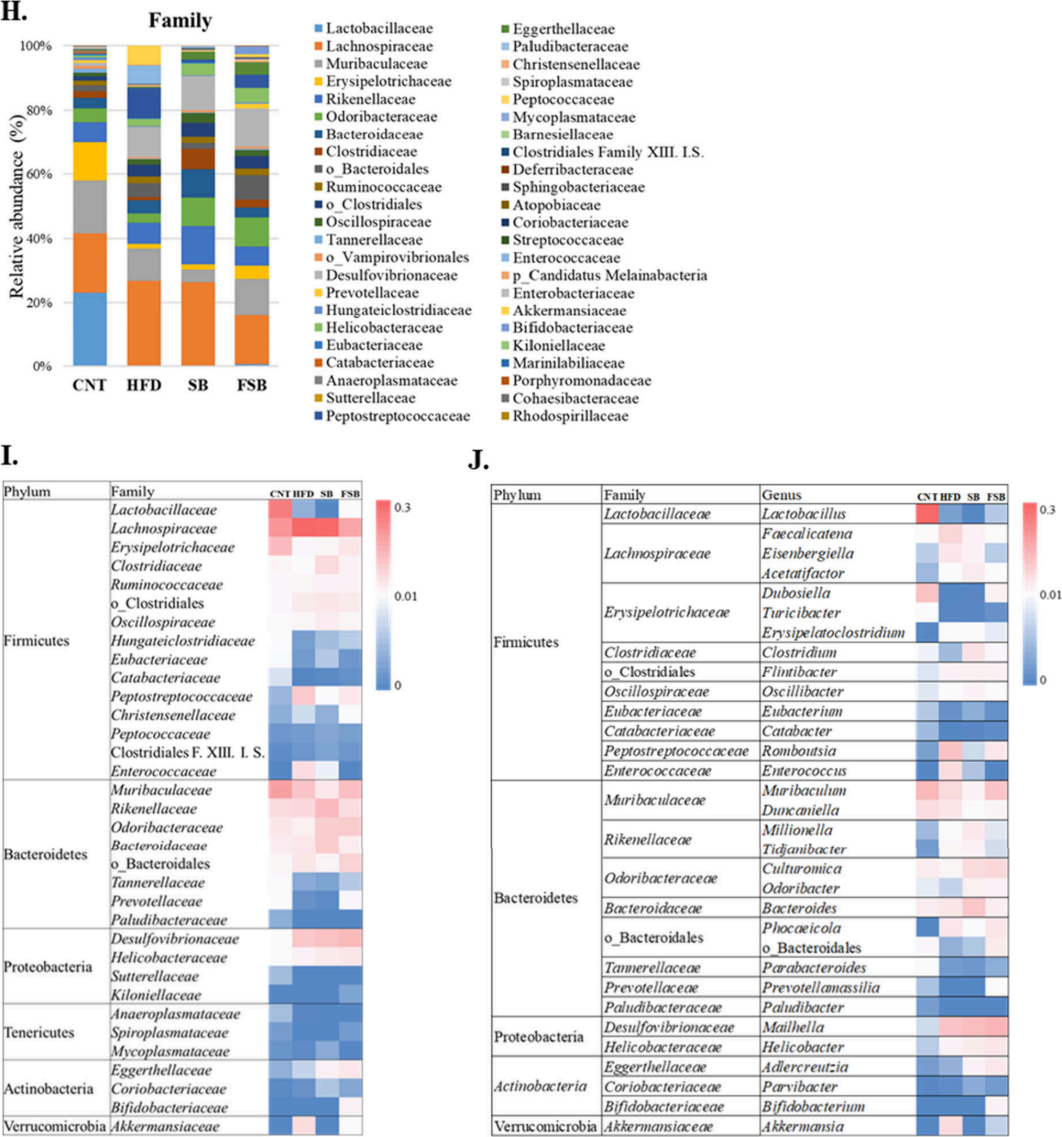
Fecal metagenomic analysis from stool samples recollected at the end of the experiment on the different mice groups (4 mice per group): control (CNT) and subjected to a high-fat diet (HFD), supplemented with soy beverage (SB) or supplemented with fermented soy beverage (FSB). (A) Shannon index (α diversity) of fecal microbiota, (B) fecal microbial taxa composition at the phyla level, (C) Firmicutes relative abundance by treatment, (D) Bacteroidetes relative abundance by treatment, (E) Firmicutes/Bacteroidetes ratio (F/B), (F) Proteobacteria relative abundance by treatment, (G) Actinobacteria relative abundance by treatment, (H) fecal microbial taxa composition at the family level, (I) heatmap of the relative abundance of the most representative families, and (J) heatmap of the relative abundance of the most representative genera.

The analysis at the phylum level showed important differences (Figure 6B). The high-fat diet decreased the relative abundance of Firmicutes compared to the CNT group, but only the SB and FSB groups exhibited a significant reduction (panels B and C of Figure 6). The difference in Bacteroidetes suggests a decrease in the HFD against the CNT group, although there was not statistical significance, restored in the groups with the intake of soy beverages (Figure 6D). Hence, according to the mentioned results of these phylums, the Firmicutes/Bacteroidetes (F/B) ratio was lower in the SB and FSB groups, with only the F/B ratio being from FSB samples significantly lower than those from HFD and CNT groups (Figure 6E). Contrary to the previous observations, the high-fat diet increased the relative abundance of Proteobacteria, independent of the intake of any soy beverage (Figure 6F). However, the presence of Actinobacteria was significantly increased in the samples from the groups supplemented with both soy and fermented soy beverages compared to the CNT (Figure 6G).

At the family and genus levels (panels H, I, and J of Figure 6), several differences were observed between the microbiota of the four experimental groups. A high-fat diet caused an alteration of the representatives of Firmicutes in HFD samples through the dramatic reduction of Lactobacillaceae (*Lactobacillus*) and Erysipelotrichaceae (*Dubosiella* and *Turicibacter*) and the increase of Lachnospiraceae (*Faecalitena* and *Esisenbergiella*), Peptostreptococcaceae (*Romboutsia*), and Enterococcaceae (*Enterococcus hirae*) (panels I and J of Figure 6). As for the Proteobacteria, the HFD group showed the disappearance of Prevotellaceae (*Prevotellamassilia*) and Paludibacteraceae (*Paludibacter*) and a decrease in Tannerellaceae (*Parabacteroides*). Moreover, Desulfovibrionaceae (*Mailhella massiliensis*) and Akkermansiaceae (*Akkermansia muciniphila*), belonging to Proteobacteria and Verrucomicrobia, respectively, increased their abundance in a HFD (Figure 6I).

Supplementation of a soy beverage during the high-fat diet caused similar changes in the relative abundance of Firmicutes and Proteobacteria in the SB group to those observed in HFD, although a slight decrease of Peptostreptococcaceae and Enterococcaceae families and an increase in Clostridiaceae (*Clostridium*) was observed (panels I and J of Figure 6). With regard to Bacteroidetes, SB showed differences with a HFD in an increment of Rikenellaceae (*Millionella* and *Tidjanibacter*), Bacterioidaceae (*Bacteroides*), and Odoribacteraceae (*Culturomica* and *Odoribacter*). The Eggerthellaceae and Coriobacteriaceae families, belonging to the Actinobacteria phylum, increased in the SB group due to *Adlercreutzia* and *Parvibacter cecicola*. Similar to CNT, no evidence of Akkermansiaceae was found in the feces from the SB group (Figure 6I).

Finally, the supplementation with the fermented soy beverage further reduced the abundance of the families of the phylum Firmicutes due to the additional lowering of the Lachnospiraceae family in comparison to the other two groups with a high-fat diet, although it minimally recovered the CNT levels of *Dubosiella* and the lack of *Enterococcus*. Bacteroidetes in FSB showed a decrease of Rikenellaceae (*Millionella* and *Tidjanibacter*) compared to the increased levels in HFD and SB and the increment of Prevotellaceae. Although the abundance of Desulfovibrionaceae and Helicobacteraceae was similar to those of HFD and SB, FSB was characterized by the highest representation of Eggerthellaceae (*Adlercreutzia*) and Bifidobacteraceae (*B. pseudocatenulatum*) (panels I and J of Figure 6).

## Discussion

Diets with high-fat consumption have numerous harmful effects on health, including obesity and the development of pathologies, such as type 2 diabetes, cardiovascular diseases, and a decrease in fertility.^22,23^ Therefore, it is imperative to explore solutions to prevent obesity, reduce body fat, and improve the lipid profile. Previously, a soy beverage fermented by *B. pseudocatenulatum* INIA P815 was tested on a model of perimenopause and menopause in mice, showing beneficial effects on the lipid profile and fertility of female mice,^10^ thus prompting its selection for this study. Additionally, in this work, the soy beverage was supplemented with flaxseed extracts containing lignans and flavonoids.^3^ This soy beverage supplemented with flaxseed and fermented by *B. pseudocatenulatum* INIA P815 was applied to a mouse model of obesity induced by a high-fat diet while also investigating its effects on fertility. An increase in the bioactive forms of lignans, isoflavones, and other flavonoids was observed in the fermented beverage due to the metabolism of *B. pseudocatenulatum* INIA P815. Hence, this resulted in an increase in the bioactive forms of flavonoids and lignans in the plasma (daidzein, genistein, SECO, and herbacetin), liver (daidzein, genistein, and ENL), and ovary (daidzein, genistein, and SECO) of mice that ingested the fermented beverage.

In the same way, an increase in the antioxidant activity of both fermented and non-fermented beverages and plasma from mice that consumed these beverages was observed. The antioxidant activity of the beverages used in this work was much higher than that found in the fermented and non-fermented soy beverages used in a previous study.^10^ This greater antioxidant activity could be attributed to supplementation with flax extracts rich in lignans and flavonoids.

In this work, it was possible to verify how the long-term consumption of a high-fat diet was associated with an increase in the percentage of accumulated WAT, the size of adipocytes, and the total blood cholesterol levels, although the weight of the animals did not show a significant increase. It has been reported that, in certain cases, when the mouse follows a diet with high levels of fat or energy, animals reduce the intake of food as a way to regulate the high energy content.^24,25^ In this study, the daily energy (kcal) intake was very similar in the four groups, independent of the diet. Proportional weight of the gonadal fat pad has been generally accepted as a simple reliable estimate of total body fat in normal or obese mice.^26^ Other measurements, such as relative white (WAT) and brown (BAT) adipose fat, could be also considered as well as changes in the size of adipocytes.^13^ In our study, the percentage of relative ovarian, interscapular, and inguinal WATs increased significantly in the HFD, SB, and FSB groups as well as the size of adipocytes compared to the CNT group. Although the beverages did not have an effect on the percentage of WAT pad weight, a reduction in the mean area of adipocytes was observed in the histological sections of ovarian WAT from the SB and FSB groups in comparison to the HFD group. In this way, both beverages had an influence on the size of the adipocytes of the ovarian adipose tissue, reducing the increase in their size when a diet rich in fat was provided. In this aspect, it has been described that the administration of flaxseed lignans caused a decrease in adipocyte cell volume and fat accumulation, although significant differences in body weight were not found.^27,28^ Similarly, the administration of genistein has been described to inhibit the adipose lipid deposition in mice under a high-fat diet^5,29,30^ and to reduce lipogenesis in adipocytes,^31^ while daidzein has show *in vitro* an increment of lipolysis in adipocytes.^32^ A smaller dose of lignans and isoflavones in the present work compared to those could account for the lack of effect in reducing the WAT percentage in the SB and FSB groups, while a reduction in the adipocyte size was recorded. In this work, the beverages were administered besides the diet to mimic a daily intake of a vegetable beverage, while in those others, the pure compounds were added by mixing with the diet by oral gavage or even through injections, hence achieving higher doses. Moreover, genistein shows controversial results regarding adipogenesis because it has both an adipogenic effect in some *in vitro* studies through the activation of peroxisome proliferator-activated receptor γ (PPARγ) but also the contrary effect and the reduction of lipid accumulation *in vivo* that could be mediated by the activation of estrogen receptors (ERs).^33,34^ Consumption of a high-fat diet also leads to the deposition of fat in the liver and insulin resistance.^35,36^ Despite this, the mice from the HFD, SB, and FSB groups did not present an increase in liver weight or lower glucose tolerance. Thus, the effect of flavonoids and lignans associated with improving glucose tolerance and insulin resistance could not be observed in this model.

The fermented soy beverage ameliorated the effects of a high-fat diet on the plasma lipid profile. Plasma total cholesterol increased significantly in the three groups fed a high-fat diet, but the cholesterol levels achieved in the FSB group were lower than those achieved in the HFD and SB groups. This is in accordance with the decrease in total cholesterol levels observed in mouse and rat plasma due to the presence of aglycones in fermented soy beverages.^37,38^ Likewise, the flaxseed lignans can lower plasma cholesterol.^39,40^ Furthermore, the mice in the FSB group registered high concentrations of herbacetin in plasma; a compound that has been described to have antihyperlipidemic effects, reducing plasma cholesterol levels in mice under a high-fat diet.^36^ Contrary to what was expected, the groups that received a high-fat diet suffered a decrease in plasma total triglyceride levels. Various studies show that, in obesity and following a high-fat diet, the expression of genes involved in lipid metabolism can be modified.^41–43^ In relation to this, Qiao et al.^41^ verified that obese mice suffered a decrease in blood triglycerides during pregnancy due to the reduction in the expression of angiopoietin-like protein 4, a lipoprotein lipase inhibitor. Moreover, plasma lipid levels in postprandial blood samples can be altered in obese mice due to an abnormal accumulation of triglycerides in the enterocytes and a reduction on the rate of their excretion in chylomicrons after ingestion, although clearance of triglycerides in enterocytes is achieved during fasting.^44^ That could be occurring in this case, as the blood samples were not collected after the fasting period. HDL-C levels decreased in all groups fed with a high-fat diet, and these levels did not increase when the tested beverages were administered; however, other studies show how an increase in HDL-C occurs upon administration of a fermented soy beverage with probiotics,^45^ which could be related to an increase in genistein.^46^

Impact of a high-fat diet in the intestinal microbiota is of relevance due to the role of the microbiota in the regulation of energy absorption, appetite, fat storage, chronic inflammation, and circadian rhythms.^47^ A high-fat diet and obesity have been linked to a decrease in the microbiota diversity and the increment in the F/B ratio of the microbiota, due to an increment in Firmicutes and a decrease of Bacteroidetes.^48,49^ Nevertheless, we did not find significant differences in diversity, and those fila showed opposite tendencies in the groups under a high-fat diet. Therefore, in a HFD, Firmicutes showed an increment of Lachnospiraceae that has been described as responsible for the increment of the F/B ratio,^50^ alongside Peptostreptococcaceae and Enterococcaceae, while in SB and especially FSB, those increments were less marked. On the contrary, in the three groups under a high-fat diet, Firmicutes suffered a huge decrease in Lactobacillaceae and Erysipelotrichaceae, the other two most abundant families in this phylum in the CNT microbiota. That resulted in a significant lowering of Firmicutes in the groups receiving soy beverages and a significant reduction of F/B in the FSB, which is in accordance with a possible ability of polyphenols to reduce the F/B ratio.^49,50^ With regard to Bacteriodetes, the most abundant families showed small changes with a high-fat diet, while families like Prevotellaceae, Paludibacteraceae, and Tannerellaceae decreased or even disappeared. A different response to a high-fat diet of certain Firmicutes families could be in part explained by the selective pressure that higher levels of bile acids exert on the gut microbiota;^51^ this fact could explain the increase of Lachnospiraceae, which is characterized by its ability to metabolize primary bile acids and produce acetate.^52,53^ On the contrary, the sudden reduction of Lactobacillaceae could be linked to the unequal distribution of active bile salt hydrolases within the *Lactobacillus* genus.^54,55^ It is worth noting that some studies have shown opposite results regarding the increment of the F/B ratio with a high-fat diet,^56–59^ which can be influence by other factors as well other phyla, such as Proteobacteria.

Administration of both beverages augmented the abundance of Actinobacteria, particularly, the genus *Adlercreutzia* in both SB and FSB groups and *B. pseudocatenulatum* in FSB, with the latter presumably due to its administration in the fermented beverage and with ability to transform phenolics and produce SCFAs.^60,61^ SCFAs play a crucial role on the effects that microbiota and diet exert on the host, including effects on lipid metabolism, intestinal barrier, inflammation, and immune response.^62,63^ Changes in microbiota and/or fiber content as result of a long-term high-fat diet could end in a significant decrease in total SCFA, which could be related to the pathogenesis of metabolic syndrome.^64^ Previous studies have shown a significant decrease in total SCFA and acetic, propionic, and butyric acids upon a high-fat-diet-induced obese mouse model.^65^ As expected, in our study, the three groups that followed a high-fat diet suffered a decrease in SCFA levels, being possibly related to the marked decrease of *Lactobacillus* in those groups, while other SCFA-producing microbial groups,^62,66^ such as *Roseburia* or *Eubacterium*, did not show differences among the mice groups. However, the FSB group showed moderate higher levels of SCFAs than the other two receiving the high-fat diet, which is agreement with the presence of *Bifidobacterium* in the fermented beverage and the microbiota.^61,67–69^ On the contrary, *Bacteroides* and *Clostridium*, which are also among the microbiota groups related to SCFA production,^63,70^ were increased in the SB groups, but no increment on the SFCA levels was observed. SCFAs are one of the proposed mechanisms by which the intestinal microbiota can exert an effect on cholesterol levels, lowering the synthesis rate and plasma levels,^62,71–73^ which is in agreement with the amelioration of TC levels in the FSB group.

Finally, the fermented soy beverage could be favoring the percentage of blastocyst/female mouse, an indicator of mice fertility, compared to those fed with the non-fermented beverage, showing an improvement in mice fertility upon consumption of the fermented beverage, as seen in previous studies.^10^ These beneficial effects could be attributed to the presence of *B. pseudocatenulatum* INIA P815 and the fermentation process, which produce more bioavailable and bioactive compounds, achieving a greater impact on health. The fermented soy beverage was found to be rich in isoflavone aglycones and other bioactive compounds, thanks to the β-glycosidases of *B. pseudocatenulatum* INIA P815.^9^ For decades, there have been several studies that have talked about the unfavorable effects that phytoestrogens have on fertility.^74^ However, some studies show that isoflavones does not pose a danger in reproduction.^75,76^ In the other hand, the mother’s intestinal microbiota directly affects the growth of the fetus and placenta; in this way, it has been seen that *Bifidobacterium* would favor placental structure, nutrient transport, and fetal growth.^77^ Furthermore, there is a relationship between SCFA derived from maternal intestinal microbiota and embryo quality by improving embryo survival.^78^ In this study, following a high-fat diet did not have a negative effect on the fertility of female mice. Various studies verified that overweight female mice did not negatively affect spontaneous ovulation and natural fertilization; however, the pre-implantation of fertilized ovules and the development of blastocysts and embryos was affected.^79^ In our study, we observe a higher percentage of blastocysts per female upon consumption of the fermented beverage in female mice, showing a beneficial effect of this beverage on the fertility in this animal model. This is the first time that the effect of this kind of beverage has been tested on the fertility of female mice under a high-fat diet. In women, a relationship between obesity and infertility has been demonstrated.^80–82^ Therefore, the development of functional products, such as our fermented soy beverage with lignans, could offer additional benefits on fertility, compensating for the negative effects of a high-fat diet.

To summarize, the soy beverage fermented by *B. pseudocatenulatum* INIA P815 and enriched with lignan extract demonstrated significant health benefits compared to the unfermented soy beverage in various parameters studied in a mouse model under a high-fat diet. Mice receiving this fermented beverage exhibited higher concentrations of flavonoids and lignans in their bioavailable form in the liver and plasma, accompanied by an increase in antioxidant activity in the latter. Hence, these mice experienced an amelioration on the rise of total plasma cholesterol levels and adipocyte growth caused by a high-fat diet. Furthermore, the fermented beverage regulated their intestinal microbiota and influenced the improvement in SCFA production. Finally, the fertility of mice ingesting the fermented beverage improved compared to mice receiving the non-fermented beverage, reaching a higher percentage of blastocysts per female mouse. Thus, the fermented soy beverage demonstrated potential in promoting mouse health, ameliorating to some extent the effects of a high-fat diet.
